# The Gibbs Paradox and Particle Individuality

**DOI:** 10.3390/e20060466

**Published:** 2018-06-15

**Authors:** Dennis Dieks

**Affiliations:** History and Philosophy of Science, Utrecht University, P. O. Box 85.170, 3508 AD Utrecht, The Netherlands; d.dieks@uu.nl

**Keywords:** Gibbs paradox, identical particles, distinguishability, Bose-Einstein, Fermi-Dirac

## Abstract

A consensus seems to have developed that the Gibbs paradox in classical thermodynamics (the discontinuous drop in the entropy of mixing when the mixed gases become equal to each other) is unmysterious: in any actual situation, two gases can be separated or not, and the associated harmless discontinuity from “yes” to “no” is responsible for the discontinuity. By contrast, the Gibbs paradox in statistical physics continues to attract attention. Here, the problem is that standard calculations in statistical mechanics predict a non-vanishing value of the entropy of mixing even when two gases of the same kind are mixed, in conflict with thermodynamic predictions. This version of the Gibbs paradox is often seen as a sign that there is something fundamentally wrong with either the traditional expression S=klnW or with the way *W* is calculated. It is the aim of this article to review the situation from the orthodox (as opposed to information theoretic) standpoint. We demonstrate how the standard formalism is not only fully capable of dealing with the paradox, but also provides an intuitively clear picture of the relevant physical mechanisms. In particular, we pay attention to the explanatory relevance of the existence of particle trajectories in the classical context. We also discuss how the paradox survives the transition to quantum mechanics, in spite of the symmetrization postulates.

## 1. Introduction

The Gibbs paradox has two versions, thermodynamic and statistical. The thermodynamic paradox is that the mixing of two different ideal gases leads to an increase of the total entropy that is independent of the nature of the gases that are being mixed. For example, in the paradigm case of two equal amounts of different gases, both at temperature *T* and in an original volume of size *V*, the entropy growth resulting from the removal of a partition between the gases is 2Nkln2 (with *N* the number of molecules of each of the gases and *k* Boltzmann’s constant). However, this entropy of mixing suddenly drops to zero when the two gases are equal, which conflicts with the intuition that small changes should have small effects.

By contrast, the statistical Gibbs paradox is that application of standard formulas from statistical mechanics leads to the prediction of a growth of entropy of 2Nkln2 even when two ideal gases of the same kind are mixed. It therefore appears that, in this case, statistical mechanics does not attain its goal of reproducing the phenomenal facts. Many have held that the paradox has a deep significance and that its solution requires a change of either the statistical expression for the entropy or a fundamental change in the statistical counting methods.

In this article, we first recall the essential role of pragmatic considerations in the solution of the thermodynamic Gibbs paradox. We then show how similar considerations hold the key to the solution of the statistical paradox, without a need for changes in basic notions of the orthodox Boltzmannian approach. Along the way, we shall discuss and sometimes criticize some of the proposed alternative solutions. The quantum theory of “identical particles” will turn out to be only marginally relevant: even in the case of quantum systems, the Gibbs paradox persists—it is merely the numerical *value* of the mixing entropy that is affected by quantum considerations. The present discussion is more comprehensive than in our earlier papers on this subject [1,2,3,4], adds new considerations both in the classical and quantum contexts, and corrects inaccuracies.

## 2. The Gibbs Paradox in Thermodynamics

Consider a fixed amount of ideal gas in a container with volume *V*, in contact with a heat bath. The macroscopic, thermodynamic state of the gas is in this case determined by the pressure *P* and temperature *T*. The quantity $\int_{1}^{2}\delta Q/T$, i.e., the supplied heat divided by the temperature, integrated along a reversible path from state 1 to state 2 turns out to be independent of the path between 1 and 2—this is one of the experimental pillars of thermodynamics. It follows that a state function, the *entropy* *S*, can be defined such that $S\left(P_{2},T_{2}\right)-S\left(P_{1},T_{1}\right)=\int_{1}^{2}\delta Q/T$. Use of the ideal gas law to evaluate the integral leads to the following expression for an ideal gas (*N* is the fixed number of molecules):

(1) $$S\left(P,T\right)=\frac{5}{2}Nk\ln T-Nk\ln P+C,$$

in which *C* is independent of *P* and *T*, but could depend on other things like the nature of the gas. It is evidently natural to take the same value of *C* for different systems consisting of the same gas, and also to take the total entropy of two isolated systems as the sum of the individual entropies. These conventions (in the sense of choices not being forced upon us by direct experimental facts) make the entropy *additive* (this argument follows ([5], p. 305).

Now, consider an adiabatic (δQ=0) and reversible removal of the partition between two volumes of the same ideal gas (For this, we must assume the absence of surface effects, adsorbing walls, etc.). This procedure of enlarging the original systems defines the *N*-dependence of the entropy, and we conclude that the entropy is *extensive*: before the removal of the partition the total entropy was twice the entropy of each of the individual systems by virtue of additivity, and the removal of the partition does not change the total entropy. Extensivity implies:

(2) $$S\left(P,T,N\right)=\frac{5}{2}Nk\ln T-Nk\ln P+cN,$$

in which *c* does not depend on *P*, *T* and *N*. Equation (2) tells us how *S* varies with *N* when particles flow in or out of the system. Evidently, it would have been impossible to fix this *N*-dependence had we confined ourselves to the study of the closed systems to which Equation (1) applies—that equation contained *N* not as a variable but as a fixed number.

An essential premise in the derivation of Equation (2) was the assumption that the adiabatic removal of a partition between two volumes of the same gas (with equal *P* and *T*) led to a *reversible* process of mixing. However, when a partition is removed between two volumes of *different* ideal gases, this results in an *irreversible* process in which the two gases rapidly expand into the greater volume, so that the entropy increases even if the process is adiabatic. Therefore, we cannot use the above procedure for computing the resulting entropy when two different gases mix. However, it is also possible to let different gases mix in a reversible way, by using the device of semi-permeable membranes, transparent to one gas but not to the other. If these membranes are slowly moved, each of the two gases reversibly expands into its final volume. We can calculate the amount of heat that has to be supplied in order to keep the temperature constant while the gases are performing work on their respective semi-permeable membranes by using the equations of state of the two gases. For two equal initial volumes *V* of different ideal gases, each with the same *P* and *T* and each expanding into the same final volume 2V, the increase of entropy is found to be:

(3) $$S_{mix}=2kN\ln2.$$

The value of this entropy of mixing is independent of the difference between the two gases, so it remains the same when (in thought) we make the gases more and more similar. This is the thermodynamic Gibbs paradox.

The reason that the entropy of mixing does not gradually disappear is readily understood from the above. The derivation of Equation (3) had as its main premise that the two gases can be reversibly mixed (and separated) by some device that is able to fully distinguish the molecules of one gas from those of the other (the “semi-permeable membranes”). This assumption was made regardless of how small the differences between the gases were, and regardless of how difficult it is in practice to construct such devices. This assumption of complete separability was only abandoned in the case of chemically equal gases, in which there is no thermodynamically relevant difference that could be used for a separation procedure, and in which Equation (2) applies. The discontinuous drop from 2kNln2 to 0 thus reflects the discontinuous step from treating gases as separable or not, a step from “yes” to “no”.

As has been addressed by Grad [6], Jaynes [7], van Kampen [5] and others (compare also [8]), the discontinuity in question does not need to correspond to the presence or absence of a fundamental physical difference between the two gases. An experimenter who does not possess the technical means to separate two gases that are theoretically different (according to present-day insights, or perhaps according to a yet to be discovered theory) will not run into empirical contradictions when he or she maintains that there is no mixing at all, and no mixing entropy. The consequences of a non-vanishing mixing entropy, in terms of heat or work, can only become empirically relevant via the use of techniques that actually make a difference between the gases. In the absence of such procedures, or the interest to use them, the gases can be taken to be equal from a pragmatic point of view. The situation thus is completely perspicuous and intelligible.

## 3. The Gibbs Paradox in Statistical Mechanics

One of the fundamental ideas of statistical mechanics is that the thermodynamic entropy relates to the number of microstates compatible with a given macrostate. In what I will call the Boltzmannian tradition, this idea is combined with a dynamical randomness assumption, so that the second law of thermodynamics becomes understandable as a consequence of probability considerations: as a rule, a physical system will evolve from macroscopic states with fewer micro realizations to macrostates corresponding to more microstates, with the result that the entropy grows. The standard formal expression of this relation between entropy and microstates is

(4) S=klnW,

with *W* the number of microstates associated with the macrostate with entropy *S*. As in the case of the thermodynamic entropy, there will be no empirical consequences when a constant is added to the entropy of Equation (4), in which case we would obtain S=klnW+C .

To see what Equation (4) implies about the *N*-dependence of *S*, consider two equally-sized containers with volume *V*, filled with the same ideal gas in equilibrium at *P* and *T*, with an insulating wall between them. Equation (4) tells us that the total entropy possesses twice the value of the individual entropies (since the total number of accessible microstates is the product of the numbers of microstates of the two individual systems), so that the entropy defined in (4) is additive. When we remove the partition between the two volumes, however, Equation (4) does not predict that the entropy will remain the same and that the combined system will therefore possess twice the entropy value of each of the original single systems. Instead, if the number of microstates in each of the individual volumes *V* was *W*, the number of microstates in the combined volume would not be $W^{2}$ but rather

(5) W22NN=W22N!N!N!,

in which the binomial coefficient represents the number of ways the 2N molecules can be distributed over the two volumes when the partition is no longer in place (the molecules originally on the left now also have access to the right compartment, and vice versa).

Within the orthodox Boltzmannian approach, associated with the ergodic hypothesis and its modern successors, the presence of the combinatorial factor is justified by the fact that it makes a (micro) difference *which* particle is *where* in the total particle configuration. For this, it must make sense to say that, e.g., a particle that originally was located on the left later finds itself on the right. In other words, the particles are assumed to possess an *individuality*, in the sense of identity over time, which makes it physically meaningful to attach labels to them that are preserved over time. Classical physics indeed provides such an identity marker, namely the trajectory of each individual particle. The trajectory connects the locations of a particle over time and thus defines what it means to speak about the same particle at different instants. Without this existence of trajectories, a physical notion of individuality would need some other physical marker that persists in time. In the philosophical literature, non-physical notions of “primitive thisness” or “haecceity” have also been proposed for this purpose. However, such metaphysical concepts are not needed given the existence of classical trajectories and, needless to say, do not play a role in physical theory.

The thus defined individuality of classical particles is responsible for the existence of a difference between instantaneous microstates of an ideal gas (consisting of particles of the same kind) that relate to each other via particle exchanges. It makes a difference, for example, whether a particle at a certain location originally came from the left or from the right—these are different microstates. After the removal of the partition in the Gibbs set up, macrostates can therefore be realized in more micro ways than before: the accessible phase volume has grown.

The formula S=klnW leads to the following entropy value for the combined system after mixing (Stirling’s approximation has been used for the factorials, which is justified since we are interested in the thermodynamic limit of very large *N* values):

(6) $$2k\ln W+k\ln\frac{2N!}{N!N!}=2k\ln W+2kN\ln2.$$

An increase of entropy therefore appears, with the familiar value of Equation (3). The same entropy increase is found if we start with two volumes *V* filled with *different* ideal gases and let them mix. Because the origin of this entropy increase lies in the growth of the phase volume accessible to the individual gas molecules, it does not disappear in the case of gases of the same kind.

We have thus hit on what in the Boltzmannian approach is the essence of the statistical Gibbs paradox: according to statistical mechanics, viewed as a theory that relies on the notion of individual particles that keep their identity over time, the entropy grows as a result of mixing, even when there is no entropy increase according to thermodynamics. The statistical entropy calculated from Equation (4) is therefore not extensive, although the thermodynamic entropy is (cf. [4]).

## 4. Proposed Solutions of the Statistical Paradox

The traditional response to the discrepancy that thus arises between the statistical and thermodynamic entropies consists in adjusting the definition of the statistical entropy by dividing the value of *W* in Equation (4) by N!. Instead of the original S=klnW for each of two volumes of the same ideal gas, before mixing, we now obtain S=klnW/N!, while the entropy of the combined system should be divided by 2N! and thus becomes

kln{2NNW2/2N!},

which is exactly double the entropy of each of the original volumes. Thus, introducing the additional factor 1/N! in all entropy expressions makes the statistical entropy extensive and removes the statistical Gibbs paradox.

As just presented, this appears an *ad hoc* modification of the definition of entropy, introduced with the mere purpose of conforming to the thermodynamic results. In order to have a *solution* of the Gibbs paradox, we would instead need an *explanation* for the insertion of 1/N! that should provide physical understanding of why the factor 1/N! is needed. In the literature, two strategies that attempt such a justification of 1/N! have prevailed: one grounded in the thought that the individuality and therefore distinguishability in principle of classical gas molecules has not been taken into proper account in the standard calculations, and the other—the dominant view—based on the idea that gas particles of the same kind should be considered as *permutable*, in the sense that an interchange of any two of them does not change the state, so that we have overcounted the number of possible microstates. In the latter approach, a final justification is sometimes sought in the way “identical particles” are dealt with in quantum mechanics. We shall discuss these two approaches in turn (The discussion in this section elaborates and improves on Sections 4 and 5 of [3]).

### 4.1. The Statistics of Particles Possessing Individuality

The Gibbs paradox is about the release of a constraint, so that gas molecules that were confined to a fixed region become free to move into a bigger volume. For the statistical treatment, we have to consider the new microstates that become available.

Consider again the standard example of an ideal gas in equilibrium in a volume of size *V* that is brought into contact with a gas of the same kind and in the same macrostate, in a volume of the same size *V*. Suppose that, before the removal of the partition *W* micro states were available to each of the two gases. As discussed in the previous section, the new number of micro states in the total system is W2(2NN): each way of dividing the total number 2N of particles into two distinct groups of size *N* corresponds to another total particle configuration. As we have seen in Section 3, this way of counting is based on the assumption that each particle possesses its own individuality. For the sake of illustration, suppose that at some initial instant (with the partition in place) the particles in the left compartment are numbered 1,2,…,N, and the particles on the right side N+1,N+2,…,2N, and also that exactly *N* locations are available in each of the two compartments. In this case, the number of microstates on each side before the removal of the partition is N!, and therefore the initial number of states in the total system $N{!}^{2}$. After the removal of the partition, there are exactly 2N locations available to 2N particles, so that the number of microstates of the total system has grown to (2N)!. This illustrates the increase of the number of microstates by the factor (2NN) (In a realistic illustration, we should of course consider a number of possible locations that is much greater than the number of particles. This complicates the calculation because in this case we have to take into account (non-equilibrium) situations in which the particles are not evenly distributed over the total volume; they could, for example, all be in the left compartment. The probability consideration that follows also covers this case.).

The basic assumption in the Boltzmannian approach to statistical mechanics is that all microstates of the same energy have an equal probability of being occupied (This is justified by the ergodic hypothesis or one of its modern successors—this is a subject in itself, which we are not going to discuss). If we adopt this assumption, the probability of having $N_{1}$ particles in the left compartment and $N_{2}=2N-N_{1}$ particles in the right one (with 2N the fixed total number of particles) is given by the binomial distribution

(7) P(N1,N2)=2NN1122N=2N!N1!N2!122N.

The particle numbers $N_{1}$ and $N_{2}$ have now become stochastic variables; it has been assumed that on average each ideal gas particle finds itself equally often in the left and right compartments so that the probabilities of being on the left or right are 1/2. Note the appearance of the factors $1/N_{1}!$ and $1/N_{2}!$ in Equation (7). These come from the binomial factor (NN1), whose presence is justified by the assumption that it makes a difference *which* individual molecules out of the total collection find themselves in the volumes $V_{1}$ and $V_{2}$, respectively. The probability in Equation (7) is proportional to the size of the phase volume of the total system (as a function of $N_{1},N_{2}$), so that we may take the logarithm of $P(N_{1},N_{2})$ as the entropy of the system.

The appearance of the factors $1/N_{1}!$ and $1/N_{2}!$ in Equation (7) is suggestive: as we have seen in the beginning of this section, insertion of such factorials makes the entropy expressions extensive and dissolves the Gibbs paradox.

The relation with extensivity is made more explicit by the following consideration. If we have two volumes of different sizes, $V_{1}$ and $V_{2}$, and let the total particle number and also $V_{2}$ go to infinity, while the particle density remains finite and constant (the thermodynamic limit), we find for the limiting probability that $N_{1}$ particles are in $V_{1}$:

$$P\left(N_{1}\right)=K\frac{{V_{1}}^{N_{1}}}{N_{1}!},$$

in which *K* is a constant. The entropy of an *N*-particle system with volume *V* in contact with an infinite particle reservoir therefore becomes (*N* is the particle number, fluctuating around the equilibrium value at which it is sharply peaked):

(8) $$S=\ln\frac{V^{N}}{N!}+\mathrm{constant}.$$

This essentially is the derivation of the *N*-dependence of the entropy given by the grand canonical ensemble (the grand canonical ensemble characterizes systems in contact with a heat bath and an infinite particle reservoir). The resulting entropy is extensive, in the sense that it varies linearly with the particle number when this number fluctuates due to particles flowing in and out of a particle reservoir.

However, in the Gibbs set up, there is no external particle bath, but only two finite gas volumes that are brought into contact with each other but whose combination is assumed to be isolated from the rest of the world. If the initial two compartments were each in open contact with particle reservoirs to begin with, the situation would change and we would no longer have the original Gibbs problem (However, even in the case of open systems in contact with infinite particle reservoirs, a variation on the Gibbs paradox can be reconstructed ([5], p. 309), in spite of the occurrence of the factorials in the grand canonical expressions). Thus, let us see what the predictions are of Equation (7), with its factorials, if it is used directly for the actual Gibbs case. In particular, we are interested in the question of whether the factorials in Equation (7) provide the extensivity needed to solve the paradox.

Immediately after the removal of the partition (an infinitesimal time interval later, say), the particles 1,2,…,N are still on the left and the other particles are still on the right (with the labeling as introduced above); according to the binomial distribution, this configuration has a probability of ${\frac{1}{2}}^{2N}$ (there is only one way of dividing the total collection of particles in these two groups). When the mixing has taken place, there will be a non-vanishing probability for all possible particle distributions (including the ones in which one of the compartments is empty). However, since the binomial probability is very sharply peaked around $N_{1}=N_{2}=N$, with overwhelming probability an equilibrium will be established in which the gas is (practically) uniformly distributed over the total volume. The associated change in entropy can be expressed as the logarithm of the new probability, which, according to Equation (7), is:

(9) ▵S=kln2NN=2kNln2.

If we start with two volumes *V* filled with *N* atoms of ideal gas *A* and *N* atoms of gas *B*, we find the same value for the entropy of mixing. This was to be expected because, on the particles microlevel, exactly the same dynamical processes, with the same probabilities, will take place in the two cases. Thus, even in the case of gases of the same kind, we obtain a non-vanishing entropy of mixing, if we compute probabilities on the assumption that the particles possess individuality.

There is, however, a way to avoid this conclusion, namely by arguing that, in the case of gases of the same kind, the situation immediately after the removal of the partition already *is* a state of maximum probability and maximum entropy because the particle density on both sides *already has* the equilibrium value at which the probability peaks. This would lead to the result (desirable from the thermodynamic point of view) that mixing of gases of the same kind (and same *P* and *T*) does not result in a growth of entropy.

However, note that this way of avoiding the statistical Gibbs paradox does not rely on the occurrence of the factors 1/N! in the entropies as justified by the binomial probability. The conclusion that there is no entropy of mixing in the case of gases of the same kind is here due to *our decision not to distinguish* between the original situation in which the initial left and right particles were in their home compartments and the later situation in which the molecules have randomly redistributed themselves. This is a decision to disregard microscopic differences. By contrast, the probability Formula (7) *presupposes* the relevance of precisely such differences, by assuming that the permutation of particles leads to a new situation—this assumption motivated the N! in Equation (7). Forgetting about the microscopic differences actually goes *against* the philosophy of individual particles that lies at the basis of Equation (7); not taking these differences into account is therefore a purely pragmatic decision (i.e., motivated by our interests rather than by fundamental physical aspects of the situation).

The pragmatic nature of the argument does obviously not at all imply that the disregard of particle details is *unjustified*: evidently, when we are focused on the prediction of macroscopic thermodynamic quantities, it makes no sense to bother about micro differences. The point we make is that this solution of the Gibbs paradox is unrelated to the microscopic details of the situation, and independent of the appearance of the factors 1/N! (in the binomial distribution) that is due to these microscopic details.

Another way of arriving at the same conclusion is by noting that the probability distribution of Equation (7) cannot tell us how the entropy of the combined system depends on the total particle number—this number 2N is constant in the Gibbs situation. Therefore, we cannot derive from Equation (7) that a factor 1/(2N)! should appear in the total entropy. This means that the traditional solution of the paradox mentioned at the beginning of this section—namely: divide the numbers of states of the partial systems by N! *and* divide the total number of states by (2N)!—cannot be justified by applying Equation (7) to the Gibbs situation [2,4]. For suppose that we define the total entropy as suggested in the discussion above, following Equation (7) ([5,9], see also [10]):

(10) $$S\left(N_{1},N_{2}\right)=k\ln P\left(N_{1},N_{2}\right)+C=k\ln\frac{1}{N_{1}!}+k\ln\frac{1}{N_{2}!}-k\ln\left(2^{N}\right)/\left(N!\right)+C,$$

with a constant *C* that can be arbitrarily chosen. Then, it is true that a choice for *C* can be made such that the resulting formula *suggests* a total entropy that is extensive: choose $C=k\ln2^{N}/N!$ and we find

$$S\left(N_{1},N_{2}\right)=k\ln\frac{1}{N_{1}!}+k\ln\frac{1}{N_{2}!},$$

in which the total entropy is the sum of two partial entropies (In the more general case of unequal partial volumes $V_{1}$ and $V_{2}$, we would arrive at the entropy expressions $S_{i}=k\ln{\left(V_{i}/V\right)}^{N})/N_{i}!$, with *V* the total volume). However, making the entropy linear in *N* in this way would be achieving extensivity *by fiat*, by the conventional choice of a different constant *C* for each individual value of *N*. This extensivity by choice clearly does not give us an explanation on the basis of what physically happens on the microlevel. Of course, it was not to be expected that we can derive a physical *N*-dependence of the combined system because this system is isolated and *N* is constant. Therefore, the factorials in Equation (7) do not imply extensivity of the combined system and do not solve the statistical paradox (Swendsen [11] has proposed an approach that formally looks similar, starting from Equation (7) and entropy as the logarithm of the probability, but with the important difference that the probability in Equations (7) and (10) is interpreted in an information theoretic sense, namely as a representation of our uncertainty about where individual particles are located [12]. Swendsen argues that the form of the dependence of the entropy on *N* can in this case be derived even for closed systems: since we are ignorant about *which* particles, from all particles of the same kind in the world, are located in the system in question, the probability formula Equation (7), with the desirable factor 1/N!, applies. From a Boltzmannian point of view, this information theoretical argument about particles in other systems cannot yield a physical explanation for what happens in the isolated Gibbs set up. In [13,14], Swendsen responds to criticism, but does not address our concerns).

Summing up, taking account of the individuality of classical particles in the orthodox approach justifies the use of binomial coefficients and therefore factors of the form 1/N! in the probabilities; however, this does not imply the extensivity of the entropy when two gases of the same kind are mixed and does not solve the statistical Gibbs paradox (This is not to deny, of course, that the grand canonical ensemble, with its factor 1/N! in the probability distribution as derived from the binomial distribution, plays an essential role in problems in which particle numbers can vary, for example in the study of dissociation equilibria [9]. What we deny is that this grand canonical factor is relevant for the solution of the Gibbs paradox).

### 4.2. The Effects of Particle Permutability

The traditional justification for inserting a factor 1/N! in the entropy goes back to Gibbs himself and to Planck (see [5,9,15] for references to the early literature) and relies on the argument that we have overcounted the number of states because a permutation of particles of the same kind does not change the state. In the case of a system consisting of *N* particles of the same kind, we must accordingly divide the number *W*—obtained by traditional counting—by N!, the number of permutations of *N* particles.

In order to judge this argument, we need to be clear about the intended sense of “exchanging particles” (cf. [4], Sections 2 and 3). If a permutation of conventionally chosen particle *labels* is intended, permutability is a truism. Empirical facts concerning a particle system are completely determined by physical particle properties, and names or labels that do not represent such physical features are irrelevant; this holds independently of whether the particles have the same intrinsic properties or not. This trivial character of label permutability makes it irrelevant for the Gibbs paradox. We need a more substantial notion of exchangeability if it is to be physically relevant.

The exchange notion implicit in most permutability arguments seems to be the following. Consider particles of the same kind (i.e., with the same intrinsic properties), suppose particle 1 is in the one-particle state *a*, and particle 2 is in the state *b*. Now take, not as a concrete physical process but merely in thought, particle 1 with its intrinsic properties and substitute it for particle 2, in the state *b* particle 2 was in; and, vice versa, put particle 2 in the state *a* first occupied by particle 1. Since the two particles have the same intrinsic properties, nothing has changed in the physical situation after this swap—except for the interchange of the labels 1 and 2 that attach to the particles. Since these labels are conventional, we can change them back (leaving the particles where they are), so that we end up in exactly the same situation as when we started. In this way, we obtain $N_{i}!$ permuted states of particles of kind *i* with exactly the same physical properties. To eliminate this superfluous multiplicity, we can divide the total number of states *W* of a system consisting of $N_{i}$ particles of kind *i* by the factor $\Pi_{i}N_{i}!$, so that we obtain a phase volume that is smaller than the volume considered before permutability was taken into account. With Equation (4), the new counting method leads to a reduced value of the entropy: $k\ln(W/\Pi_{i}N_{i}!)$. As we have seen, this is exactly the expression needed for extensivity of the entropy and disappearance of the statistical Gibbs paradox.

This justification for dividing by $N_{i}!$, and thus for passing from ordinary state space to the “reduced state space”, is convincing if the exchanges are not associated with physical differences. The case of a particle interchange as just described is an example of such an unphysical change: here, the swapping of particles of the same kind was a mere mental operation and not a physical process. The intuitive appeal coming from the term “particle exchange” is deceptive in this context: it is obscure if there is anything exchanged at all. In the case of particles of the same kind, placing particle 1 in the state of particle 2, and vice versa, only makes sense if the particles possess an identity over and above their intrinsic physical properties and states. In philosophy, individuating principles of this sort are sometimes discussed (“primitive thisness” or “haecceity”), but such concepts are not recognized in physical theory. In accordance with this, statistical mechanics is not meant to consider situations as different that relate to each other by the permutation of putative non-physical particle identities. This seems to entail that, in the case of particles of the same kind, we should never use the usual unreduced state space but ought to always pass to the reduced state space where all states differing by exchanges of particle labels have been collapsed into one.

However, the use of the *unreduced* state space for particles of the same kind is legitimate if the particle labels (which also number the coordinate axes of the unreduced state space) can be defined in physical terms. In the Gibbs set up, we may define such physically meaningful labels in the following way. Number the particles, in the initial state, according to their positions—for example, from left to right on the basis of their horizontal spatial coordinates. Thus, the particles in the left compartment receive the labels 1,2,…,N, and the particles on the right-hand side are numbered N+1,N+2,…,2N. Now, it is important that, although these assignment of labels is conventional, once given, the labels stick to the particles over time via the trajectory that each particle follows. Thus, given this initial labeling, it makes sense to say that, at some later point in time, particles 1 and N+1 may end up in states *x* and *y*, respectively, but that it may also be the other way around (1 in *y* and N+1 in *x*); and that these two states differ from each other because of the individuality of the particles. In the first case, the particle that originated from the left-hand corner of the container occupies state *x*, and, in the second case, it is another particle that does so. If particle labels are defined in this way, for particles of the same kind, there surely is a physically defined difference between “particle *i* at *x*, particle *j* at *y*” and “particle *i* at *y*, particle *j* at *x*”; and this distinction is in principle because it may be that the difference is practically irrelevant, in which case we may revert to the reduced phace space—see the discussion at the end of this section) relevant to the calculation of *W*. The numbering of axes of the unreduced state space, in cases where it is physically significant to use the unreduced phase space, should be definable in exactly this way: they must refer to physically defined individuality markers. What an interchange of two particle labels in this case captures is not that two duplicates of a state can be imagined by mentally swapping metaphysical particle identities, but rather that there are two different physical cases, a real physical swapping of particles, that lead to two distinct situations. This multiplicity of states plays a role in the orthodox calculation of probabilities, via the ergodic hypothesis or one of its modern successors, and there is therefore no a priori justification for discarding the differences through division by N!.

In a *closed* system, even such physically understood multiplicities are unimportant for the calculation of entropies, though, because, for each microstate, the *same* factors $N_{i}!$ appear in the number of ways it can be realized. This factor therefore drops out in the probability expression P=(number of microstates with propertyA)/(total number of microstates), which means that all empirical predictions of statistical mechanics remain the same when we divide by $\Pi_{i}N_{i}!$ [4]. In this case, the division is a harmless cosmetic operation, which can also be seen from the fact that S=klnW implies that all entropy values only change by the constant additive term $-k\ln\Pi_{i}N_{i}!$.

For systems that are subject to external manipulation, in the sense that something is done to the system that affects the number of possible particle trajectories, the situation is different, though. In the Gibbs case that starts with two equal volumes of equal gases, both with initial particle number *N*, the multiplicity of realizations of any microstate with the partition in place is ${\left(N!\right)}^{2}$ because the particles cannot move out of their compartments (the *N*-particle states localized in each individual compartment can each be realized in N! possible ways). After removal of the partition, the multiplicity of states with *N* particles on both sides becomes much greater: now, we must take into account that the particles may go from one side to the other. As a result, after the removal, there are (2NN) different ways that the particles can distribute themselves over the total volume.

There are therefore many more evolutions and states that lead up to macroscopic equilibrium than before: the only originally allowed situations, in which particles stayed in their own compartments, have become statistical oddities. Given any initial state just before the partition was removed, the probability is overwhelming that particles will move out of their original regions and will redistribute themselves approximately uniformly over the total volume. The total amount of phase volume available to the system has grown spectacularly, which is measured by the additional mixing entropy kln(2NN)=2Nln2. Because classical particles always possess identity over time, the calculation of the mixing entropy remains the same regardless of whether the particles have the same intrinsic properties or not.

Summing up the results of these two subsections, the statistics of particles possessing individuality does not entail that the entropy is extensive in the Gibbs set up. Quite the opposite, individuality is the essential factor responsible for the appearance of an entropy of mixing: particles have their own individual trajectories according to classical physics, and the possibility of physical exchanges of particles leads to a growth of microstates and consequently to an increase of the statistical entropy.

Evidently, the growth in accessible phase volume that is at issue here will more often than not be without empirical consequences because its detection involves the identification of individual particle paths (if there are no chemical differences between the gases). This introduces a notion of *pragmatic non-individuality and permutability of particles*. If we consider the difference between gas particles coming from the left and right as immaterial in practice (as we must do by definition if we are only interested in macroscopic quantities), there is no practical point in thinking of an increase of the phase volume. In the numbers of states bookkeeping, we can in this case divide all multiplicities after mixing by (2NN) (expressing that it does not matter from which compartment the particles originally came; we factor out the associated multiplicity)—this removes the entropy of mixing. This procedure gives us the right empirical entropy values, given the measurement limitations inherent in thermodynamics (In [8], the authors present an elegant general information theoretic account of how entropies on different levels of description relate to each other. It follows from their treatment that ignoring particle individualities and trajectories leads to the appearance of a factor 1/N! in the entropy expression, in accordance with what we argue). The reduction of the number of states and the transition to the reduced state space is thus certainly justified, but we should not conclude that the unimportance of trajectory information for the usual phenomenal predictions implies the non-existence of differences on the microlevel (Saunders [16], by contrast, takes the position that microstates really and literally remain the same, as a fundamental microscopic fact, when two particles of the same kind are swapped. According to his analysis, the absence of an entropy of mixing is due to this fact. This is a major difference with our argument).

## 5. Quantum Mechanics

In quantum mechanics, it is a basic principle that states of particles of the same kind (i.e., with the same intrinsic properties) must be completely symmetric or anti-symmetric under permutations. In the case of bosons, we have symmetrical many-particle states that do not change under permutations, whereas the states of fermions are anti-symmetrical, thus incurring a minus sign under uneven permutations.

To see exactly what is permuted, and what the consequences are, consider first the classical two electrons case again. According to the usual (unreduced) phase space representation as we have discussed and defended it in Section 4.1, if the electrons are in the states ϕ and ψ, respectively, the total system is in one of two states, $\phi_{1},\psi_{2}$ or $\phi_{2},\psi_{1}$, where the labels refer to some distinguishing property that persists over time. As we have noted, distinctions on the basis of such labels may be practically irrelevant so that we may switch over to the reduced classical phase space. In quantum mechanics, the (anti-)symmetrization postulates tell us to do something similar, but now not as a pragmatic choice but as a law-like principle: states solely differing by a permutation of the indices are combined into one superposed state. In the case of two quantum electrons, this results in an anti-symmetrical total state, e.g.,:

(11) $$|\Psi \rangle =\frac{1}{\sqrt{2}}{(|\phi \rangle }_{1}{|\psi \rangle }_{2}-{|\psi \rangle }_{1}{|\phi \rangle }_{2}).$$

In an *N*-particles state, the N! product states (The multiplicity is N! if each one-particle state occurs only once, as is the case for fermions; for bosons, we may assume the same in the thermodynamic limit) that are permuted copies of each other are similarly united in one (anti-)symmetrical superposition. Consequently, instead of N! possibilities, we have only one state. This reduction of the number of states seems highly relevant for the Gibbs paradox: it provides us with a factor 1/N! at exactly the right place, as instead of the classical value S=klnW (with *W* the number of states of labeled particles) we now obtain S=klnW/N!, not as a pragmatic choice but as a matter of principle. According to this line of thought, the interchange of two particles from the left and right, respectively, does not lead to a new state and therefore not to a new physical situation, so that there can be no entropy of mixing in the case of two equal gases.

However, there are some caveats here; and taking them into account undermines the just-mentioned conclusion. First, it is important to realize that the permutations considered in the symmetrization postulates permute the *indices* that occur in the *N*-particle state. These indices refer to the individual Hilbert spaces whose tensor product forms the *N*-particles Hilbert space, but do not label what we ordinarily would call “particles” in the classical limit (see [17] for an extensive discussion of this point). For example, in a case in which the states |ϕ〉 and |ψ〉 in Equation (11) represent two narrow and well-separated spatial wave packets, it is these *wave packets* (so *states*) that correspond to “electrons” as identified in laboratory practice. These individual wave packets approximately follow classical particle trajectories (In this special case of wave packets with distinguishable trajectories, quantum mechanics describes entities that are like individual classical objects. In [16,18,19], Saunders considers such emerging “individuals” as new objects, which differ from quantum particles; the latter he takes to correspond to non-identifiable (“weakly discernible”) objects. In our opinion, it is better to say that, in these cases, *particles* emerge as individual entities, and that the concept of a particle is not always applicable at the fundamental quantum level. One among several reasons was already noted: the “individuals” correspond to what are identified as particles in laboratory practice [1,4,17,20]; another that the “weakly discernible” objects are always in exactly the same state, so that they can never possess individuating properties. For example, all electrons (conceived as indistinguishables) in the universe possess exactly the same spatial and non-spatial characteristics. It seems odd to call such putative entities “particles”). By contrast, the two indices 1 and 2 in Equation (11) are both associated with exactly the same physical state, namely the density operator $\frac{1}{2}(|\phi \rangle \langle \phi |+|\psi \rangle \langle \psi |)$. Thus, any physical entities labeled by these indices are in exactly the same state, which implies that the permutation of 1 and 2 is without physical meaning. However, that does not follow at all for particles that are defined by the individual orthogonal states |ϕ〉 and |ψ〉. *These* particles can be distinguished, and in special situations they mimic the behavior of individual classical particles with trajectories.

There is a second important ingredient to be added to the argument in order to make the analogy with the classical situation closer. In the initial Gibbs situation, it is assumed that the two groups of particles are dynamically confined to their own compartments. This means that there must be interactions between the walls (including the partition) and the particles. In the case of narrow wave packets, these interactions can be visualized as collisions between the particles (as identified by the wave packets) and the walls. This implies that the total quantum state will not be as in Equation (11) (or its many-particles analogue), but must include the environment. In any realistic model of the interactions, the particles will leave their imprints on the walls and this implies that a sort of physical labeling of the particles takes place: the environment keeps a record of the particles. This point is reinforced when we also consider what must happen when we try to separate particles originating from the two compartments by means of the quantum analogues of semi-transparent membranes. These membranes should be devices that are sensitive to the difference between wave packets coming from the left and right, respectively; and should be able to respond differently in these two cases. This would lead to a detection and effectively a physical labeling of the particles.

To include this additional structure in the expression for the total Gibbs state, we have to introduce states referring to the environment. Let the interaction at some early stage between electron state |ϕ〉 and initial environment state $|E_{0}\rangle$ lead to the environment state $|E_{a}\rangle$, and interaction with |ψ〉 to the environment state $|E_{b}\rangle$. Thus:

$$\begin{array}{ccccccc} \left|\phi \rangle \right|E_{0}\rangle & ⟶ & \left|\phi \rangle \right|E_{a}\rangle & \mathrm{and} & \left|\psi \rangle \right|E_{0}\rangle & ⟶ & \left|\psi \rangle \right|E_{b}\rangle . \end{array}$$

Then, instead of Equation (11), we obtain:

(12) $$|\Psi_{tot}\rangle =\frac{1}{\sqrt{2}}(|\phi \rangle |E_{a}\rangle |\psi \rangle |E_{b}\rangle -|\psi \rangle |E_{b}\rangle |\phi \rangle |E_{a}\rangle ).$$

In this formula, the different environment states correlated with |ϕ〉 and |ψ〉 , respectively, serve as physical markers of these electron states. Now, in order to see whether the removal of the partition in the Gibbs set-up results in a growth of the available state space, we can repeat the argument of Section 4.1. We can write for the initial state instead of Equation (12):

(13) $$|\Psi_{tot}\rangle =\frac{1}{\sqrt{2}}{(|\phi \rangle }_{a}{|\psi \rangle }_{b}-{|\psi \rangle }_{b}{|\phi \rangle }_{a}).$$

Now suppose that, after the partition has been removed, there is a possible state with one electron in state $|\phi^{\prime }\rangle$, on the left, and one in state $|\psi^{\prime }\rangle$, on the right. Then, there are two possibilities for the total state (and different evolutions leading up to these states), namely

(14) $$|\Psi_{tot}^{\prime }\rangle =\frac{1}{\sqrt{2}}{(|\phi^{\prime }\rangle }_{a}|\psi^{\prime }\rangle_{b}-|\psi^{\prime }\rangle_{b}|\phi^{\prime }\rangle_{a}),$$

and

(15) $$|\Psi_{tot}^{\prime }\rangle =\frac{1}{\sqrt{2}}{(|\phi^{\prime }\rangle }_{b}|\psi^{\prime }\rangle_{a}-|\psi^{\prime }\rangle_{a}|\phi^{\prime }\rangle_{b}).$$

These are two different two-particles states (The physical meaning of these states can be made more transparent by using the formalism of “wedge products” [21]. With the wedge product ∧, the states (14) and (15) assume the forms $|\phi^{\prime }\rangle_{a}\wedge {|\psi^{\prime }\rangle }_{b}$ and $|\psi^{\prime }\rangle_{a}\wedge {|\phi^{\prime }\rangle }_{b}$, respectively, with the natural interpretation of individual particles *a* and *b* in switched states). The difference between these states corresponds, just as in the classical case, to a *physical interchange* between the two particles. The situation is essentially the same as the one of two individual classical particles that follow definite trajectories and switch positions: |ϕ〉 has developed into $|\phi^{\prime }\rangle$ and |ψ〉 into $|\psi^{\prime }\rangle$, or |ϕ〉 has become $|\psi^{\prime }\rangle$ and |ψ〉 has become $|\phi^{\prime }\rangle$. One of these two states was not possible before the removal of the partition, and has become so afterwards. The number of possible states has thus grown by a factor 2; in the general *N*-particles case, the multiplicity is N!.

In some situations, the (anti-)symmetrical quantum states therefore show characteristics that are very similar to those of classical *N*-particle states, so that analogous considerations apply concerning the Gibbs paradox. A Maxwellian quantum demon would be able to distinguish wave packets coming from the left from those coming from the right, and verify the existence of an entropy of mixing.

Admittedly, situations in which the quantum states mimic the behavior of classical particles are very special cases. However, also in more general situations, a distinction in principle between one-particle states originally coming from the right or left is possible. Each one-particle state that is localized in the right compartment (in the initial Gibbs situation) is orthogonal to all one-particle states in the left compartment. This mutual orthogonality will be maintained by independent unitary evolution of the one-particle states (as in the case of an ideal gas). By virtue of this persisting mutual orthogonality, ideal yes-no measurements can in principle be devised that at a later stage of the evolution determine the origin of the one-particle states—needless to say that these measurements would soon become forbiddingly complicated. However, a superhumanly skillful and knowledgeable demon could exploit them to establish that there is an entropy of mixing; with the help of quantum measuring devices as analogues of semi-permeable membranes, particles originally coming from the left will be allowed to pass, others not, and vice versa. This verification of the presence of a quantum statistical entropy of mixing is completely analogous to what could be verified by a Maxwellian demon in the classical case [2]. This analogy shows that the appearance of 1/N! as a result of (anti-)symmetrization does not rule out the appearance of an entropy of mixing in the case of equal gases, and so does not solve the Gibbs paradox. This does not mean that quantum mechanics is irrelevant to the entropy of mixing. If quantum gases are not dilute (as supposed in the above arguments), Fermi-Dirac or Bose-Einstein statistics will have to be used and the multiplicity of states is no longer simply N!. This is one thing that will affect the *value* of the entropy of mixing. Another thing is the role of measurement interactions: in quantum mechanics, measurements partly determine the post-measurement properties of the measured particles. Interactions between the particles and the outside world therefore can influence the value of the entropy of mixing; this is an issue to be further explored elsewhere. There are also other complications that should be taken into account in the quantum case [22].

## 6. Conclusions

The account given in this paper is motivated by the original Boltzmannian idea to make macroscopic phenomena intelligible by interpreting them in terms of microscopic “mechanical” processes and objective probabilities (as in ergodic theory). According to this approach, the increase in entropy when two classical gases mix is a consequence of the increase in possibilities for the gas particles: they are no longer confined to one compartment but may move freely through the whole volume. Whether or not this microscopic change leads to empirical consequences depends on how discriminating our measuring techniques are. In standard thermodynamics, we only consider macroscopic features of physical systems, like pressure and temperature, and exclude the observation of individual particle paths. By contrast, chemical differences, however minute, are standardly taken to belong to the area of competence of thermodynamics. The standard thermodynamic account therefore says that there is *no* entropy of mixing in the case of gases of the same kind and that there *is* such an entropy if the gases are chemically different. However, as pointed out at the end of Section 2, it may well happen that, in an actual experiment, chemical differences are too small to be detectable, in which case they acquire the same status as hidden microscopic differences due to the origin of individual particles.

The decision to restrict ourselves to a coarse-grained level of description of what happens when gases mix is pragmatic, and it is possible to go to a deeper, microscopic level. If we do so, we should be able to verify an entropy growth even if the gases that mix are of the same kind: instead of focusing on a macroscopically accessible difference, we should in this case pay attention to a microscopic one, namely “coming from the left” versus “coming from the right” for single particles. Permutability of labels or invariance under mental particle swapping are irrelevant for this conclusion, as are arguments that derive factors 1/N! from the use of the binomial probability distribution.

The Boltzmannian microscopic explanation of these results is concretely visualizable and close to physical intuition, and in this way satisfies important philosophical standards of intelligibility [23]. There are nevertheless other ways of reproducing the thermodynamic predictions, most importantly by purely information theoretic methods. As these are less concerned with concrete physical processes on the microlevel, and use probabilities to quantify our lack of information (“subjective probabilities”), they satisfy different standards of intelligibility. Viewed from this angle, the Gibbs paradox and statistical physics in general provide an interesting case of plurality of approaches in physics.

Finally, quantum mechanics is able to describe situations that closely resemble cases from classical mechanics—a paradigm example is that of *N* narrow wave packets (coherent states) far apart in space. In such situations, quantum mechanics in very good approximation reproduces the classical predictions, and this includes the presence of an entropy of mixing. This is so regardless of the (anti-)symmetry of the total wave function, which already shows that the symmetrization postulates by themselves do not remove the Gibbs paradox. Semi-classical situations are of course an exception in quantum theory, but also, in more general quantum settings, the presence of a microscopic entropy of mixing can be expected, as briefly sketched in Section 5. It is true that quantum predictions as a rule differ from their classical counterparts, and that this also applies to the value of the mixing entropy (see the end of the previous section). However, it is wrong to think that the statistical entropy of mixing in the case of equal gases vanishes automatically because of the “identity of quantum particles of the same kind”.
